# Bilateral renal infarction in a patient with severe COVID-19 infection

**DOI:** 10.1590/2175-8239-JBN-2020-0156

**Published:** 2021-01-11

**Authors:** Percy Herrera Añazco, Fernando Mayor Balta, Liz Córdova-Cueva

**Affiliations:** 1 Hospital Nacional 2 de mayo, Departament of Nephrology, Lima, Perú.; 2 Universidad Señor de Sipán, Chiclayo, Peru.

**Keywords:** Renal Infarction, Thromboembolism, Coronavirus Infections, SARS-CoV-2, Kidney, Renal Dialysis, Infarto Renal, Tromboembolia, Infecções por Coronavirus, SARS-CoV-2, Rim, Diálise Renal

## Abstract

Thromboembolic events are frequent in patients with COVID-19 infection, and no cases of bilateral renal infarctions have been reported. We present the case of a 41-year-old female patient with diabetes mellitus and obesity who attended the emergency department for low back pain, respiratory failure associated with COVID-19 pneumonia, diabetic ketoacidosis, and shock. The patient had acute kidney injury and required hemodialysis. Contrast abdominal tomography showed bilateral renal infarction and anticoagulation was started. Kidney infarction cases require high diagnostic suspicion and possibility of starting anticoagulation.

## Resumo

Fenômenos tromboembólicos são frequentes em pacientes com infecção por COVID-19 e nenhum caso de infarto renal bilateral havia sido relatado. Apresentamos o caso de uma paciente do sexo feminino, de 41 anos, com diabetes mellitus e obesidade, que deu entrada no serviço de urgência por lombalgia, insuficiência respiratória associada à pneumonia COVID-19, cetoacidose diabética e choque. A paciente apresentava lesão renal aguda e demandava hemodiálise. A tomografia abdominal contrastada mostrou infarto renal bilateral e foi iniciada anticoagulação. Os casos de infarto renal requerem alta suspeita diagnóstica e possibilidade de iniciar a anticoagulação.

## Introduction

In December 2019, the novel coronavirus disease 2019 (COVID-19), a severe acute respiratory syndrome caused by the coronavirus 2 (SARS-CoV-2), was identified in China^1^. To date, there are more than 52 million infected people worldwide^2^ and although COVID-19 infection was initially described as a disease with respiratory symptoms, other clinical manifestations have been reported that make it a multisystemic disease^3^^-^ 5. Extrapulmonary manifestations include acute kidney injury^6^^,^ 7 and thromboembolic events^8^. Thromboembolic events in patients with COVID-19 are frequent and although the pathophysiologic mechanisms are not entirely clear, the most frequently referred thromboses are at the pulmonary and cerebral level^9^^,^ 10. The kidneys are organs susceptible to thrombosis, and evidence of thrombi at the level of glomerular capillaries has been found in necropsies of seriously ill patients^11^. Although to date some cases of patients with renal infarctions have been reported in patients with COVID-19^12^^-^ 14, these are unilateral, and to our knowledge, no case of bilateral renal infarction (BRI) has been reported. We report the case of a 41-year-old woman with severe COVID-19 infection and BRI.

## Case report

A 41-year-old woman with obesity and 6 years of diabetes mellitus without treatment came to the emergency with a history of 7 days of fatigue and 2 days of dyspnea. Additionally, she reported bilateral and abdominal low back pain that partially improved with paracetamol.

At presentation, she was hemodynamically stable, had dyspnea, tachypnea, and an oxygen saturation of 80%. Chest radiography showed bilateral basal alveolar infiltrates and the rapid test was positive for IgM against COVID-19. Chest tomography found a bilateral ground glass pattern at the bottom that occupied 35% of the lung parenchyma without signs of pulmonary embolism.

Due to an initial glycemia of 500 mg/dL, urine ketones and severe metabolic acidosis, she was diagnosed with severe metabolic ketoacidosis. The main laboratory findings are shown in Table 1.

**Table 1:** Laboratory findings of the patient

Laboratory Findings*	Patient	Normal values
Hemoglobin, g/dL	6.9	13.7-17.7
Leukocytes, ×10^3^/µL	21.8	4-10
Thrombocytes, ×10^3^/µL	25.8	150-400
PO_2_, mm Hg	83	75-100
PcO_2_, mm Hg	44	35-45
pH	7.29	7.35-7.45
FiO %	0.4	0.21
Bicarbonate, mEq/L	20	21-25
Lactate, mg/dL	0.6	5.0-15
Glucose, mg/dL	158	80-100
CRP, mg/dL	210	< 0.5
Sodium, mEq/L	130	135-145
Potassium, mEq/L	5.7	3.5-5.5
Serum creatinine, mg/dL	5.73	0.6-1.2
Aspartate aminotransferase (U/L)	36	< 35
Alanine aminotransferase (U/L)	12	< 45
**Coagulation**		
D-Dimer, ng/mL	1400	< 500
aPTT, s	30.6	25-36
PT, s	16.1	10-13
Fibrinogen, mg/dL	1036	200-400
Urinary Analysis **		
Leukocyte	0	< 5/c
Erythrocytes	7	< /3
Proteins	+	-
Ketonic bodies	+++	-
**Immunologic Analyses**		
Antinuclear antibodies	Negative	
C3 (g/L)	1.46	0.88 - 2.01
C4 (g/L)	0.45	0.16 - 0.48
Anticardiolipin IgG (GPL/ml)	Indeterminate	< 17
**Others**		
Serum homocysteine (µmol/L)	6.3	5-15
Protein C (%)	148	70–140
Protein S (%)	64	60–120
Antitrombin III (%)	124	80-120

* On the day of starting hemodialysis

** On the day of admission

CRP: C-reactive protein

aPTT: activated partial thromboplastin time

PT: prothrombin time

C3: Complement 3

C4: Complement 4

**Figure 1:**
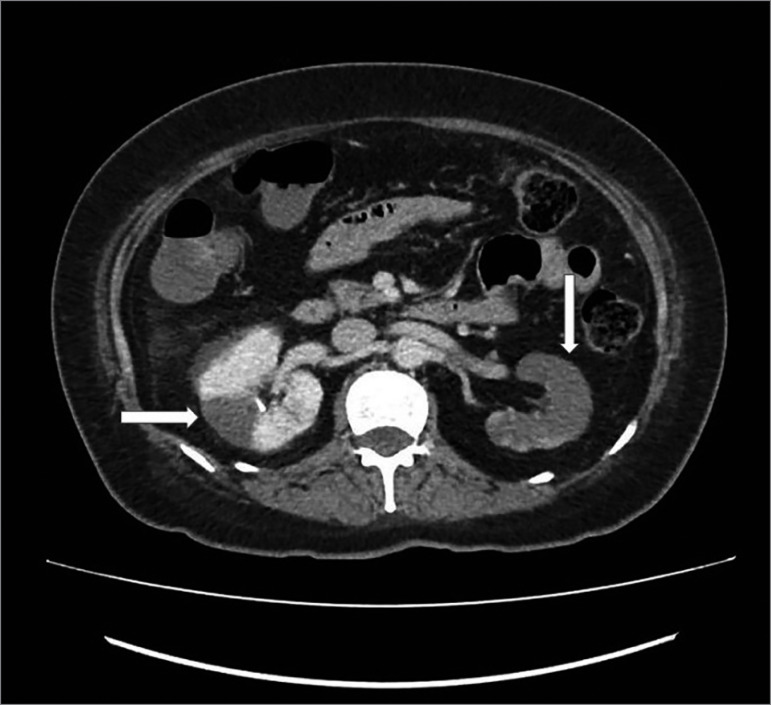
Multiple perfusion defects in both kidneys, predominantly in left kidney.

**Figure 2:**
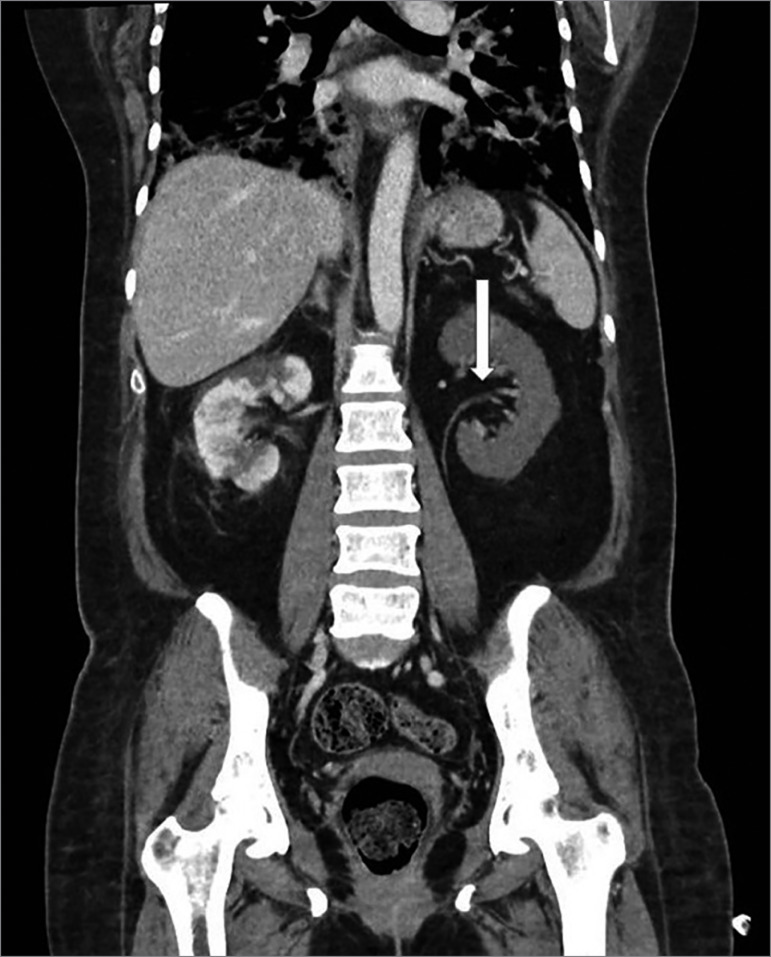
Abdominal computed tomography showing thrombus in left kidney artery immediately before its bifurcation. The thrombus totally obstructs its previous division and partially obstructs the posterior division.

Initial management included oxygen therapy, hydration with saline, insulin, ceftriaxone, dexamethasone, and ivermectin. Three days later, low back and abdominal pain worsened, and a contrast abdominal tomography was requested, which showed perfusion defects in both kidneys, predominantly in the left kidney, suggestive of renal infarctions. (Figures 1 and 2). There was no evidence of extra renal thrombosis. Due to these findings, anticoagulation was started with enoxaparin 60 mg every 12 hours. Complementary physical examination showed no signs of peripheral ischemia and electrocardiogram showed sinus rhythm. She had no past history of atrial fibrillation.

Respiratory and hemodynamic evolution of the patient was unfavorable, requiring mechanical ventilation and vasopressors. Subsequently, nine days later, the patient presented acute kidney injury (with oliguria, later anuria, and water overload) and conventional hemodialysis was started (the only type of hemodialysis available in our hospital) with non-tunneled catheter. Laboratory findings are shown in Table 1.

Two weeks later, the patient was still on hemodialysis, mechanical ventilation, and anticoagulation. She died in the third week of treatment.

## Discussion

Renal infarction is relatively rare, as an emergency department study reported renal infarction in 0.004% of visits^14^. The main cause of renal infarction is cardioembolic event in patients with atrial fibrillation, but in half of cases there is no known cause^15^. Although low back pain, renal hypertension, and acute kidney injury have been described within its clinical manifestations, it can also present with nonspecific symptoms that make its diagnosis difficult^15^.

Nearly 20% of renal infarctions are bilateral^15^, and these have been reported in cases of dissecting aortic aneurysm, septic emboli in patients with endocarditis, lupus, vasculitis, sickle cell disease, fibromuscular dysplasia of renal arteries, secondary to non-steroidal anti-inflammatory drugs, and due to suspension of anticoagulation^16^^-^ 18. The clinical presentation of BRI is more dramatic than in cases of unilateral infarction, with cases of acute kidney injury being more frequent^15^ with clinical manifestations that may even be similar to rapidly progressive glomerulonephritis^19^. Although there are no significant difference in comorbidities, patients with BRI have a worse prognosis^15^.

Although acute kidney injury in patients with renal infarction is not uncommon, especially in patients with BRI, dialysis is required only in 11% of patients with unilateral infarction^15^. Thus, we could not affirm that our patient’s acute kidney injury was exclusively associated with renal infarction because it can be multifactorial^20^. The incidence of acute kidney injury in patients with severe COVID-19 infection varies between 19 to 36% and the dialysis requirement in these patients varies between 13 to 14%^6^^,^ 7. In our patient, factors such as effect of radiocontrast, shock, and dehydration from diabetic ketoacidosis are likely to have contributed to acute kidney injury.

Although not all tests were completed for the diagnosis of other causes of renal infarction, such as screening for sickle cell and genetic origin, these represent < 7% of causes^15^. Because of the described thromboembolic events in patients with COVID-19 infection, we hypothesized that this may be the most likely cause, which is supported by suggestive findings in necropsy of patients with severe COVID-19 infection^11^. Although the causes of this association are not entirely clear, it has been suggested that renal infarction may be associated with the direct cytopathic effect of the virus on endothelial cells, the presence of proinflammatory cytokines that stimulate the expression of tissue factors, or the formation of antiphospholipid antibodies^21^^,^ 22. Because the manifestations of a renal infarction could be subclinical, the diagnosis could be incidental and underestimated. Although there are no studies evaluating the effect of anticoagulation in patients with renal infarction, the possibility of the condition should be considered^23^.

In conclusion, we present the first case of BRI found incidentally in a patient with acute kidney injury and severe COVID-19 infection. The possibility of renal infarction in such cases should be considered given the need to start anticoagulation as a therapeutic measure.
